# Obeticholic acid as an index farnesoid X receptor scaffold in metabolic dysfunction-associated steatohepatitis: a testable model for efficacy-liability separation and biomarker interpretation

**DOI:** 10.3389/fphar.2026.1879097

**Published:** 2026-08-11

**Authors:** Jinghong Liu, Xiaojuan Wu

**Affiliations:** 1 Department of Gastroenterology, The First Affiliated Hospital of Jinan University, Guangzhou, Guangdong, China; 2 Department of Gastroenterology, Ganzhou Hospital-Nanfang Hospital, Southern Medical University (Ganzhou People’s Hospital), Ganzhou, Jiangxi, China; 3 Ganzhou Key Laboratory of Gastroenterology, The Affiliated Ganzhou Hospital of Nanchang University, Ganzhou, China

**Keywords:** ACOX1, biomarker interpretation, cholesterol crystals, farnesoid X receptor, FATP5/SLC27A5, low-density lipoprotein cholesterol, metabolic dysfunction-associated steatohepatitis, obeticholic acid

## Abstract

Obeticholic acid (OCA) is not approved for metabolic dysfunction-associated steatohepatitis (MASH), and the United States development pathway for its use in precirrhotic NASH ended after regulatory non-approval. The compound is still useful scientifically, because no other farnesoid X receptor (FXR) agonist has produced a comparable human dataset in steatohepatitis. A frequent interpretive problem is that histological benefit, low-density lipoprotein cholesterol (LDL-C) elevation, pruritus and possible liver-intrinsic injury are compressed into one FXR activation continuum. This hypothesis-driven narrative article argues for a narrower reading. OCA is treated here as an index scaffold that separates clinical response, systemic lipid risk, tolerability and candidate liver-intrinsic sterol-stress biology into distinct evidence layers. We first place OCA in the current MASLD/MASH therapeutic setting, then outline hepatic and intestinal FXR pharmacology relevant to the model. Human OCA data, cross-scaffold observations from non-bile acid FXR agonists, and mechanistic work on FATP5/SLC27A5, cholesterol stress and ACOX1 are then reassessed. Within the proposed model, FATP5 is a putative OCA-linked substrate-entry node supported mainly by preclinical and humanized-transporter work. Cholesterol retention, free-cholesterol burden, cholesterol crystals, macrophage crown-like structures, IL-1beta signaling and hepatic stellate-cell activation are better handled as a candidate sterol-stress liability branch. ACOX1 is interpreted as a context-dependent modifier, not as a validated therapeutic target. The model is not a class-wide claim for all FXR agonists. It should be tested, and if necessary rejected, using matched FXR target engagement, paired human tissue and human multicellular systems.

## Graphical Abstract

**Figure:**
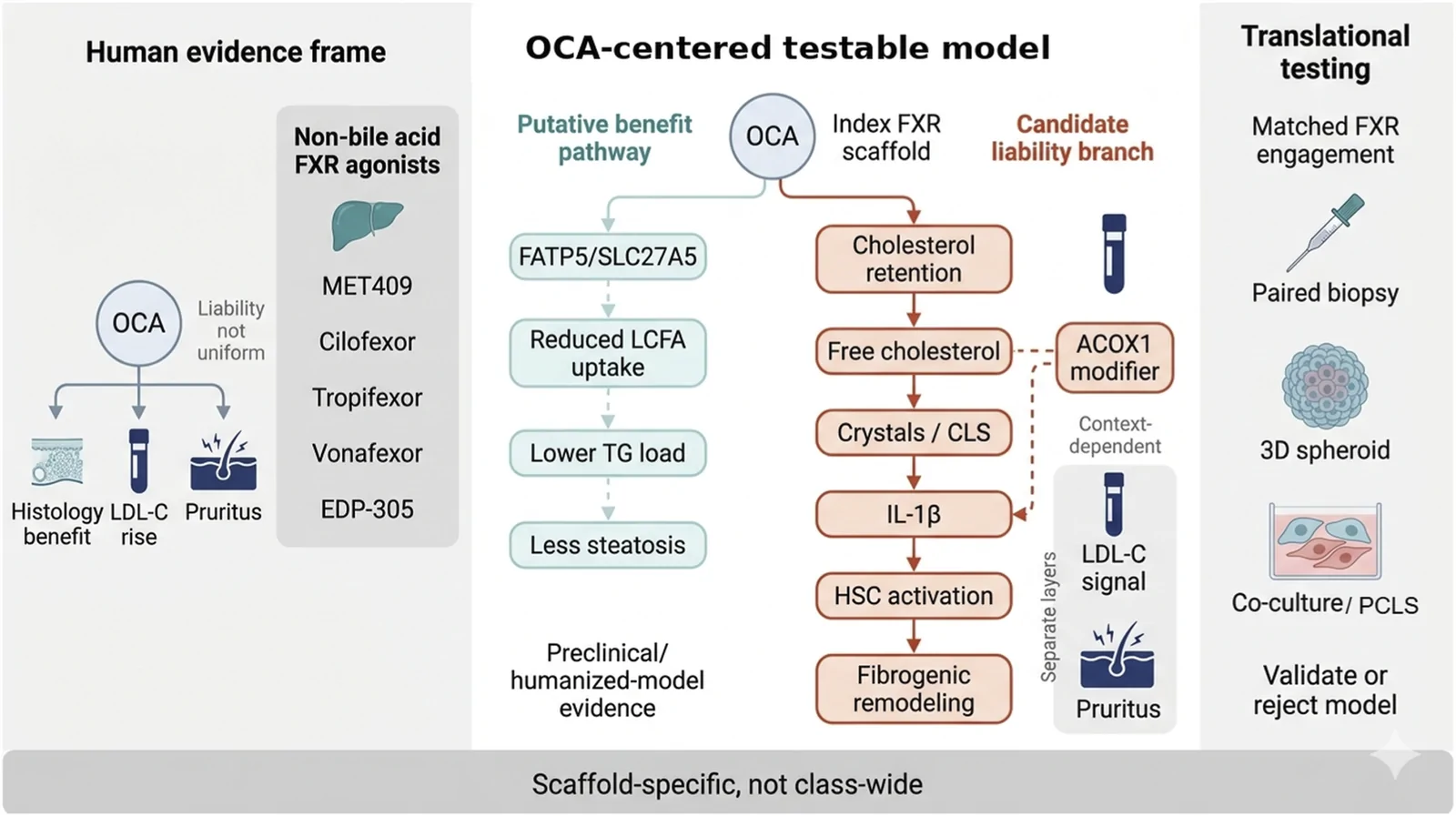
OCA is treated as an index FXR scaffold after regulatory failure in MASH. The figure summarizes the human evidence frame, the evidence-graded candidate model, and translational testing requirements.

## 1 Introduction: why OCA remains scientifically useful after regulatory failure

MASH is now being interpreted in a different clinical and regulatory landscape. The multisociety nomenclature has replaced NAFLD and NASH with MASLD and MASH, although many pivotal trials and drug-development programs were conducted under the older terms (Rinella et al., 2023a; European Association for the Study of the LiverEuropean Association for the Study of Diabetesand European Association for the Study of Obesity, 2024). The treatment landscape has also moved. Resmetirom, a liver-directed thyroid hormone receptor beta agonist, received accelerated United States approval after biopsy-based phase 3 data in adults with noncirrhotic NASH/MASH and fibrosis (Harrison et al., 2024; U.S. Food and Drug Administration, 2024). Semaglutide 2.4 mg later reported phase 3 benefit and received accelerated United States approval for adults with noncirrhotic MASH and moderate-to-advanced fibrosis (Sanyal et al., 2025; U.S. Food and Drug Administration, 2025). Tirzepatide, pegozafermin, efruxifermin and lanifibranor have widened the development field, although they differ in mechanism, maturity of evidence and regulatory status (Francque et al., 2021; Loomba et al., 2023; Harrison et al., 2023; Loomba et al., 2024). In this context, OCA is no longer best framed as a near-term MASH therapy.

OCA remains worth discussing for a narrower reason. Its development program contains metabolic, biopsy-based, lipoprotein, non-invasive biomarker, regulatory and tolerability data generated around a single FXR agonist scaffold (Mudaliar et al., 2013; Neuschwander-Tetri et al., 2015; Younossi et al., 2019; Rinella et al., 2022; Sanyal et al., 2023a). The sponsor reported a Complete Response Letter for the United States application in precirrhotic fibrosis due to NASH and discontinued NASH-directed investment (Intercept PharmaceuticalsInc, 2023). That outcome should not be equated with pharmacological inactivity. More usefully, it marks a case in which histological activity, lipid liability, tolerability and unresolved tissue-level safety questions could not be reconciled well enough for chronic treatment in a cardiometabolic population.

The difficulty is not simply the amount of evidence, but the way different signals have been grouped. Histological improvement, LDL-C elevation, pruritus and possible liver-intrinsic injury are often discussed as if they were points along one receptor-activation gradient. That reading is too coarse. Pruritus is a tolerability phenotype with bile-acid and sensory-neuronal biology, not evidence of hepatocyte injury (Alemi et al., 2013; Yu et al., 2019). Plasma LDL-C and apoB-containing lipoproteins are clinically important cardiovascular-risk variables, particularly in MASLD/MASH, but they do not demonstrate intrahepatic free-cholesterol retention, crystal formation or inflammatory-stromal activation (Pencek et al., 2016; Pockros et al., 2019; Siddiqui et al., 2020; Targher et al., 2020). The converse is also true: lower liver fat or aminotransferases cannot exclude a tissue-liability program.

The present article is therefore not another broad chronology of FXR agonist development in NASH/MASH; that ground has already been covered in recent reviews (Adorini and Trauner, 2023; Wang et al., 2024; Fiorucci et al., 2025). Nor does it use OCA as a proxy for the whole FXR agonist class. The index-scaffold framing is proposed here as an interpretive model rather than as a claim that OCA is mechanistically unique in every respect. Its purpose is to ask a more limited question: whether OCA can serve as an index scaffold for a falsifiable framework that separates clinical response, systemic lipid-risk signal, tolerability and candidate tissue branch allocation during FXR agonism in MASH.

The reason for making these categories explicit is clinical, not semantic. Patients with MASLD/MASH commonly carry obesity, type 2 diabetes, dyslipidemia, hypertension and chronic kidney disease; cardiovascular disease competes with liver-related outcomes across the disease course (Targher et al., 2020; Mantovani et al., 2021). Advanced fibrosis remains the dominant liver-related prognostic marker and is central to late-phase trial enrichment and care pathways (Kanwal et al., 2021; Rinella et al., 2023b; European Association for the Study of the LiverEuropean Association for the Study of Diabetesand European Association for the Study of Obesity, 2024). This separation is also consistent with the broader pathobiology of steatohepatitis, where metabolic stress, lipotoxicity, inflammation, hepatocyte injury and fibrogenesis overlap but are not interchangeable therapeutic or biomarker domains (Friedman et al., 2018). A drug-development framework that places lipid risk, itch, aminotransferases, liver fat and tissue injury into one undifferentiated safety column will not be very useful.

## 2 Search strategy and interpretive framework

This article was written as a hypothesis-driven narrative synthesis, not as a systematic review or meta-analysis. PubMed and regulatory-source searches were performed through May 2026 with combinations of the following terms: obeticholic acid, OCA, farnesoid X receptor, FXR agonist, MASH, NASH, MASLD, NAFLD, REGENERATE, FLINT, CONTROL, MET409, cilofexor, tropifexor, vonafexor, EDP-305, resmetirom, semaglutide, tirzepatide, FGF21, FATP5, SLC27A5, hepatic fatty acid uptake, cholesterol crystals, free cholesterol, crown-like structures, interleukin-1beta, NLRP3, hepatic stellate cell, ACOX1, peroxisomal beta-oxidation, LDL cholesterol, apoB, pruritus, MRI-PDFF, FIB-4, FibroTest, liver stiffness, FXR-SHP, CYP7A1, FGF15, FGF19, bile-acid transporters and precision-cut liver slices. Backward citation screening was used when mechanistic or pathology studies were needed to interpret a clinical signal.

The question was set before synthesis: do available data support a clinically useful distinction between anti-steatotic or histological benefit and candidate liver-intrinsic liability biology during OCA exposure? Studies were read for mechanism-bearing information, not only for whether a primary endpoint was positive. The extracted features included scaffold chemistry, population, dose, duration, target-engagement context, histological or MRI-PDFF response, lipoprotein changes, pruritus, liver injury markers, tissue cholesterol biology and the compartment in which a biomarker was measured.

Evidence was handled in four tiers. Tier one comprised OCA human intervention, lipoprotein, non-invasive marker and regulatory data. Tier two included human studies of non-bile acid FXR agonists. Tier three covered human pathology relevant to cholesterol-stress biology, and Tier four comprised mechanistic animal, cell and humanized-transporter studies. This tiering matters because a human clinical signal can define a pharmacological problem without identifying the tissue mechanism behind it. Conversely, a preclinical mechanism may be plausible yet remain unusable as a clinical biomarker. No pooled estimates or cross-trial rankings were calculated; the FXR studies differ too much in population, dosing, duration, background therapy, endpoints and tissue sampling.

Two constraints guided the interpretation. A blood biomarker was not taken as proof of a tissue mechanism without paired tissue or direct mechanistic support. Likewise, a signal observed with OCA was not generalized to all FXR agonists unless cross-scaffold human evidence pointed in the same direction. These constraints are deliberately conservative, because the proposed model is easy to overstate.

## 3 FXR pharmacology relevant to MASH: hepatic, intestinal and scaffold-specific outputs

FXR is a bile-acid-responsive nuclear receptor involved in bile-acid, sterol and lipid homeostasis (Makishima et al., 1999; Parks et al., 1999; Sinal et al., 2000). In the liver, canonical FXR activation induces SHP and represses CYP7A1, the rate-limiting enzyme in the classic bile-acid synthetic pathway, through nuclear receptor networks involving LRH-1 and related transcriptional regulators (Goodwin et al., 2000; Lu et al., 2000). FXR also regulates bile-acid transport and enterohepatic circulation, including programs involving BSEP/ABCB11 and other bile-acid handling pathways. These effects link receptor activation to bile-acid pool size, bile-acid composition, cholesterol disposal and hepatocellular stress responses (Chiang, 2004; Modica et al., 2010; Adorini and Trauner, 2023).

The intestinal arm of FXR signaling complicates any simple hepatic model. Ileal FXR activation induces FGF15 in mice and FGF19 in humans, generating an endocrine gut-liver signal that suppresses hepatic bile-acid synthesis and influences metabolic homeostasis (Inagaki et al., 2005; Jones, 2012). This is not just hepatic FXR at a distance. Intestine-specific FXR signaling has been linked to fatty liver biology through gut-derived ceramide production in preclinical models, while gut microbiota can reshape bile-acid pools that feed back onto FXR activity (Jiang et al., 2015; Sayin et al., 2013; Wahlstrom et al., 2016). A compound’s hepatic exposure, intestinal exposure, receptor potency, transporter interactions and downstream pharmacodynamic profile can therefore change the biological output even when the nominal target is identical.

This organ and scaffold dependence is central to the OCA question. OCA is a bile acid-derived FXR agonist; MET409, cilofexor, tropifexor, vonafexor and EDP-305 are structurally distinct non-bile acid or nonsteroidal agonists studied in MASH/NASH-related populations (Harrison et al., 2021; Patel et al., 2020; Sanyal et al., 2023b; Ratziu et al., 2022; Ratziu et al., 2023). Scaffold chemistry may alter receptor engagement, intestinal exposure, off-target bile-acid receptor biology, transporter interaction, pruritus, lipoprotein handling and tissue distribution. For this reason, “FXR agonist” is not a sufficiently precise mechanistic label.

Dose should be treated with similar caution. Higher exposure may increase intestinal FXR activation, bile-acid pool remodeling, pruritogenic mediator production or systemic lipoprotein effects without adding proportionate hepatic benefit. A different scaffold may achieve adequate hepatic target engagement with less intestinal or systemic exposure, but that is an empirical question rather than a class property. Dose, pharmacokinetics, bile-acid profiling, FGF19 induction and hepatic versus intestinal pharmacodynamic readouts need to be interpreted together. Exposure metrics should also be separated from nominal dose. Cmax and AUC describe systemic exposure, but they do not by themselves define liver targeting or intestinal FXR engagement. OCA illustrates this point. After multiple oral doses, the parent compound reaches peak plasma concentration earlier than its glycine and taurine conjugates, and the active conjugates undergo hepatic conjugation, biliary secretion, intestinal reabsorption and enterohepatic recirculation. In contrast, published PK/PD studies of non-bile acid or nonsteroidal scaffolds such as tropifexor and cilofexor show that systemic exposure, target engagement and tolerability cannot be inferred from nominal dose alone. Future scaffold comparisons should therefore incorporate Cmax, AUC, hepatic extraction or uptake efficiency when available, portal–peripheral exposure gradients, FGF19 induction, bile-acid profiling, C4 suppression and systemic lipoprotein changes. The therapeutic index of FXR agonists is likely governed by the relationship between portal exposure, hepatic extraction, intestinal engagement and peripheral systemic concentrations rather than dose alone (U.S. Food and Drug Administration, 2022; Badman et al., 2020; Younis et al., 2023).

The point is not that non-bile acid FXR agonists are necessarily safer or better. It is that OCA cannot stand in for the entire FXR agonist class. Any model built from OCA should remain scaffold-specific until it is reproduced under matched target engagement across chemically distinct ligands.

## 4 Human evidence from OCA development

OCA showed human metabolic activity before it entered the large biopsy-based NASH program. In patients with type 2 diabetes and NAFLD, short-term OCA treatment improved insulin sensitivity and biochemical markers, indicating measurable activity in a relevant cardiometabolic liver-disease population (Mudaliar et al., 2013). FLINT then provided biopsy-based proof of concept: OCA improved the prespecified histological endpoint more often than placebo in noncirrhotic NASH, while increasing pruritus and shifting LDL-C (Neuschwander-Tetri et al., 2015). REGENERATE extended the signal to a phase 3 fibrotic population. The interim analysis found significant improvement in fibrosis without worsening of NASH for the 25 mg dose, whereas NASH resolution without worsening of fibrosis did not reach significance (Younossi et al., 2019). A later consensus-panel analysis again supported the fibrosis signal, with one-stage fibrosis improvement without worsening of NASH in 22.4% of patients receiving OCA 25 mg versus 9.6% with placebo (Sanyal et al., 2023a).

Those results do not describe an inactive drug. They describe a biologically active scaffold whose histological signal was accompanied by reproducible lipid and tolerability liabilities. In healthy volunteers and in biopsy-proven NASH, OCA increased LDL-C or LDL particle-related measures; CONTROL showed that atorvastatin could blunt OCA-associated increases in LDL-C and LDL particle concentration (Pencek et al., 2016; Pockros et al., 2019; Siddiqui et al., 2020). For patients with MASLD/MASH, this signal is clinically relevant because cardiovascular disease contributes substantially to morbidity and mortality (Targher et al., 2020; Mantovani et al., 2021). Mechanistically, however, correction of plasma lipids with a statin does not establish that intrahepatic free cholesterol, cholesterol crystals or inflammatory-fibrogenic niches have been neutralized.

Non-invasive response markers create a parallel issue. In REGENERATE, changes in ALT, AST, GGT, FIB-4, FibroTest and liver stiffness were associated with histological response, which makes them useful for monitoring and hypothesis generation (Rinella et al., 2022). They are not spatial readouts. They cannot show whether cholesterol is retained within hepatocytes, whether crystals form in lipid droplets, whether macrophages cluster around injured hepatocytes or whether stellate cells are activated in the same microscopic niche. That gap is one reason the present model separates biomarker layers rather than treating them as interchangeable.

Pruritus also needs its own layer. In REGENERATE and related clinical experience, pruritus was frequent and dose-related, but itch should not be converted into a hepatotoxicity signal (Younossi et al., 2019). Bile-acid-induced itch has been linked to TGR5 in preclinical systems, while human cholestatic itch has been linked to MRGPRX4 (Alemi et al., 2013; Yu et al., 2019). These pathways matter for tolerability, adherence and dose selection. They do not allocate a patient to a liver-intrinsic sterol-stress branch.

The cross-scaffold evidence is imperfect but still informative. MET409 reduced liver fat over 12 weeks and did not simply reproduce the OCA profile (Harrison et al., 2021). Cilofexor, tropifexor, vonafexor and EDP-305 produced liver-fat, biochemical or proof-of-concept hepatic signals in phase 2 settings, with differences in endpoints, exposure windows and tolerability (Patel et al., 2020; Ratziu et al., 2022; Sanyal et al., 2023b; Ratziu et al., 2023). These studies cannot be ranked against FLINT or REGENERATE because many used MRI-PDFF or biochemical endpoints rather than biopsy. They do, however, make a class-wide OCA template untenable. Table 1 summarizes the cross-scaffold evidence used here.

**TABLE 1:** Quantitative clinical evidence matrix of FXR agonists evaluated in MASH/NASH.

Agent	Chemical class/context	Study design and size	Dose and duration	Main endpoint architecture	Benefit signal used here	Lipid-risk signal	Pruritus/tolerability signal	Interpretive limit
OCA	Bile acid-derived index FXR scaffold; FLINT, REGENERATE, CONTROL and lipoprotein analyses	FLINT: multicenter randomized placebo-controlled trial; REGENERATE: phase 3 randomized trial with interim and consensus-panel analyses; CONTROL: randomized lipid-management study	FLINT: 25 mg daily for 72 weeks. REGENERATE: 10 or 25 mg daily; 18-month interim histology. CONTROL: OCA with atorvastatin strategy	Biopsy histology, lipoprotein profiling, non-invasive response markers	Fibrosis-related histological signal; REGENERATE consensus-panel analysis reported one-stage fibrosis improvement without worsening of NASH in 22.4% with OCA 25 mg vs. 9.6% with placebo	Reproducible LDL-C/LDL particle rise; atorvastatin blunted plasma lipid signal in CONTROL	Frequent and dose-related pruritus in pivotal program	Strongest human FXR scaffold dataset but failed MASH regulatory path; not approved for MASH and not a class proxy
MET409	Structurally optimized non-bile acid FXR agonist	Phase 2 randomized placebo-controlled 12-week study in patients with NASH and elevated liver fat	Dose-ranging over 12 weeks	MRI-PDFF-centered liver-fat endpoint; no biopsy endpoint	Substantial liver-fat reduction over 12 weeks, with exposure-sensitive response	Differentiated LDL-C profile compared with OCA; formal cross-trial inference not possible	Exposure-sensitive tolerability; pruritus reported but not OCA-identical	Supports scaffold heterogeneity but lacks histology and direct tissue branch allocation
Cilofexor	Nonsteroidal FXR agonist	Randomized phase 2 trial in noncirrhotic NASH	Dose-ranging over 24 weeks in published phase 2 context	MRI-PDFF, biochemistry and bile-acid pharmacodynamic readouts	Liver-fat and biochemical improvement signals	Lipid profile not sufficient to define tissue sterol-stress branch	Pruritus and gastrointestinal events require dose-specific interpretation	Short-duration imaging/biochemical endpoints cannot substitute for biopsy-based OCA data
Tropifexor	Selective non-bile acid FXR agonist	Adaptive randomized placebo-controlled phase 2a/b study	Dose-ranging over short-term treatment windows	MRI-PDFF and biochemical endpoints	Anti-steatotic and biochemical signals	Lipid changes appear exposure- and dose-context dependent	Tolerability varied by dose; not a fixed class signal	Trial design differs from FLINT/REGENERATE and does not adjudicate tissue mechanism
Vonafexor	Structurally distinct FXR agonist	Proof-of-concept randomized study in individuals with suspected fibrotic NASH	Short-term phase 2 context	Hepatic and renal proof-of-concept readouts	Hepatic improvement signal outside OCA scaffold	Available data do not establish OCA-like sterol branch	Tolerability must be interpreted in study-specific exposure context	Adds scaffold diversity but cannot replace matched target-engagement comparison
EDP-305	Oral FXR agonist	Phase 2 double-blind placebo-controlled dose-ranging study in NASH	12-week dose-ranging study	ALT and MRI-PDFF endpoints	Reduced ALT and liver fat signals, particularly at higher exposure	Lipid effects require dose-specific interpretation	Dose-related tolerability limitations, including pruritus-related discontinuation in published trial context	Reinforces need to separate target engagement from scaffold-specific tolerability

## 5 Proposed model: scaffold-specific efficacy-liability separation

**FIGURE 1:**
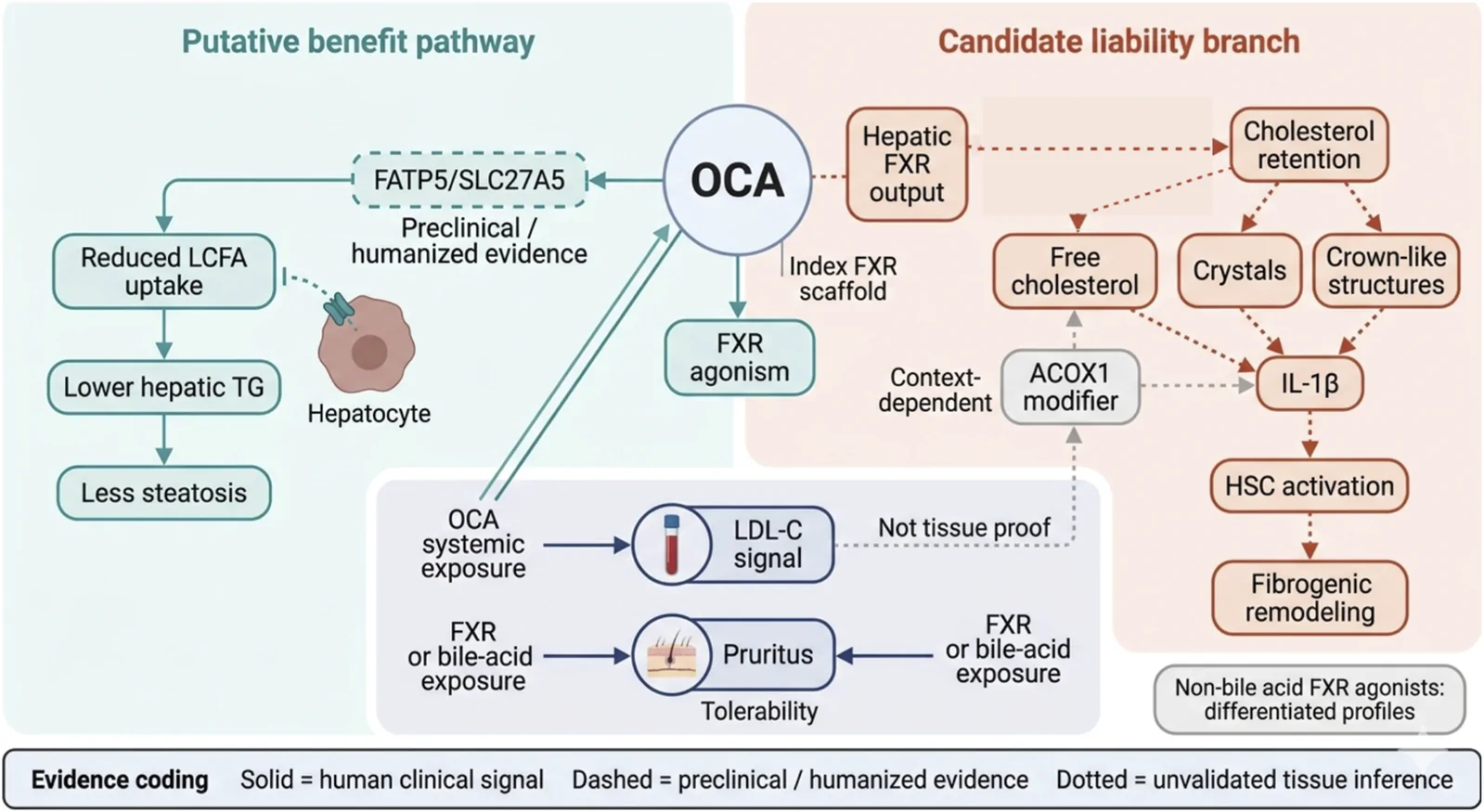
OCA-centered scaffold-specific model with evidence confidence coding. Solid arrows indicate human clinical signals, dashed arrows indicate preclinical or humanized-model evidence, and dotted arrows indicate unvalidated tissue inference. The FATP5/SLC27A5 pathway is treated as a putative OCA-linked benefit pathway, not as a class biomarker. The sterol-stress pathway is labeled as a candidate liability branch rather than a proven toxic mechanism in treated human liver.

The proposed model is intentionally narrow. It does not argue that FXR agonism as a class separates benefit from liability. Rather, it uses OCA as an index scaffold to ask whether anti-steatotic or histological benefit can diverge from systemic lipid risk, tolerability and candidate tissue-liability biology. The evidence-coded OCA-centered scaffold model is summarized in Figure 1.

The putative benefit pathway begins with FATP5/SLC27A5, but the claim should be kept modest. FATP5 is liver enriched and participates in long-chain fatty acid handling and bile acid-CoA ligase activity. Fatp5 deletion disrupts bile-acid conjugation and protects mice from diet-induced weight gain without a simple malabsorption explanation (Hubbard et al., 2006). Specific bile acids can also inhibit hepatic fatty acid uptake in mice, placing FATP5 at a plausible intersection between bile-acid chemistry, hepatocyte substrate entry and triglyceride accumulation (Nie et al., 2012). The strongest OCA-linked evidence comes from preclinical and humanized-transporter work showing that OCA inhibited long-chain fatty acid uptake more effectively in systems expressing human FATP5 than murine Fatp5 and reduced hepatic fatty acid uptake and triglyceride accumulation in FATP5-humanized models (Lin et al., 2022b).

The clinical boundary is sharp. No pharmacodynamic assay has shown FATP5 engagement by OCA in treated MASH patients. No SLC27A5-based responder biomarker has been validated in MASLD/MASH. Non-bile acid FXR agonists also show clinical signals without a validated FATP5 mechanism. FATP5 should therefore be treated as a putative OCA-linked mechanism, not as a proven mediator of clinical response or a class-level FXR biomarker.

The FATP5 statement is best written as a constrained hypothesis. Current evidence supports the possibility that FATP5 contributes to OCA-linked anti-steatotic biology in preclinical and humanized systems. It does not establish that FATP5 explains OCA’s clinical histological effect, is required for all FXR agonist efficacy, or can be used to select patients by baseline SLC27A5 expression. In figures and tables, the pathway should therefore remain coded as preclinical rather than as solid human evidence.

The candidate liability branch is better described as sterol-stress biology than as a generic toxic arm. In fatty liver mouse models, high-dose OCA produced FXR-dependent hepatotoxicity, aminotransferase elevation and fibrosis, with gene-expression changes compatible with hepatic cholesterol accumulation (Lin et al., 2022a). Species, dose and exposure differences limit direct translation to patients. Still, the pattern is not nonspecific chemical injury; it points toward sterol retention coupled to inflammatory-stromal activation.

Human MASH pathology gives this branch biological plausibility, but not proof of OCA causality. Free cholesterol is not the same as triglyceride storage. Triglyceride can function partly as a storage buffer, whereas free cholesterol can disturb membranes, mitochondria and lysosomes and can crystallize in lipid droplets (Caballero et al., 2009; Ioannou et al., 2019). Cholesterol crystals and macrophage crown-like structures distinguish steatohepatitis from simple steatosis and are strongly associated with human NASH pathology (Ioannou et al., 2013; Ioannou et al., 2019). In murine metabolic-syndrome models, cholesterol-lowering interventions dissolved crystals, dispersed Kupffer-cell crown-like structures and reversed fibrotic phenotypes (Van Rooyen et al., 2013; Ioannou et al., 2015). Cholesterol crystals can also activate NLRP3 inflammasome biology, which is relevant to IL-1beta-mediated inflammatory amplification (Duewell et al., 2010; Mridha et al., 2017; Yu et al., 2022). Macrophage-driven hepatocyte death and fibrogenesis provide a downstream stromal context in which these inflammatory lesions may translate into stellate-cell activation and matrix remodeling (Itoh et al., 2017).

None of this proves that OCA induces the same lesions in human liver during treatment. Direct evidence would require paired tissue showing that OCA exposure increases free-cholesterol stress and that sterol lesions co-localize with macrophage activation, IL-1beta signaling and hepatic stellate-cell activation. Until such data exist, cholesterol stress should remain a candidate liver-intrinsic liability branch, not an established human mechanism.

The compartment distinction is crucial. LDL-C, non-HDL-C and apoB measure plasma atherogenic particle burden and are appropriate cardiovascular-risk markers. Intrahepatic free cholesterol reflects local uptake, esterification, efflux, biliary secretion, bile-acid conversion, lysosomal handling and cell-type-specific stress responses. These compartments interact, but one does not measure the other. The proposed sterol-stress branch therefore requires liver tissue or validated human multicellular readouts; plasma LDL-C alone cannot adjudicate it.

ACOX1 is the most provisional component of the model. It catalyzes the first and rate-limiting step of peroxisomal beta-oxidation, a pathway used for lipid species that mitochondria handle inefficiently, and it generates hydrogen peroxide as part of redox signaling (Ding et al., 2021). Depending on substrate load, mitochondrial capacity, catalase-linked peroxide handling and lipid species, peroxisomal beta-oxidation may be adaptive or maladaptive. Yang and colleagues reported that pharmacological ACOX1 inhibition widened the apparent functional window of OCA in preclinical fatty liver models, improving steatosis while attenuating LDL-C, IL-1beta and alpha-smooth muscle actin signals under some conditions (Yang et al., 2024). That result is best read as preclinical profile redistribution, not as a clinical rationale for ACOX1 inhibition in MASLD/MASH.

A second scaffold-level distinction concerns hepatic–intestinal exposure partitioning. OCA, as a bile acid-derived scaffold, is expected to engage FXR within an enterohepatic cycling context, whereas structurally distinct non-bile acid agonists may differ in absorption, hepatic extraction, transporter handling, intestinal exposure and peripheral systemic distribution. This distinction matters because disproportionate intestinal FXR engagement could plausibly favor FGF19-dominant pharmacodynamic output, bile-acid pool remodeling, pruritogenic signaling and systemic metabolic effects. Conversely, stronger liver-directed engagement might preserve hepatic benefit while reducing some extrahepatic liabilities. This possibility should be treated as a testable hypothesis, not as proof that non-bile acid scaffolds are inherently safer. Current MASH trials do not provide directly comparable hepatic and intestinal tissue exposure data across scaffolds (U.S. Food and Drug Administration, 2022; Badman et al., 2020; Younis et al., 2023; Adorini and Trauner, 2023; Fiorucci et al., 2025).

Several alternative explanations must stay in view. Intestinal FXR signaling, FGF15/19 induction, bile-acid pool composition, gut microbiota, hepatic versus intestinal exposure and scaffold chemistry could all contribute to the observed benefit-liability pattern (Inagaki et al., 2005; Jiang et al., 2015; Wahlstrom et al., 2016; Li et al., 2025). Background statin therapy may blunt the plasma LDL-C signal without answering the tissue question (Pockros et al., 2019). These possibilities are not distractions from the model; they define the measurements a serious test of the model would need.

FGF19 is one of those measurements. It may mark FXR target engagement in early-phase studies, but it does not by itself identify whether the biology is hepatic, intestinal or mixed. Bile-acid composition is similar: changes in primary, secondary, conjugated and microbial bile-acid species can reflect gut-liver signaling, transporter activity and microbial metabolism. Future scaffold comparisons should therefore pair pharmacokinetics, FGF19 and bile-acid profiling with tissue-level readouts rather than relying on nominal dose.

## 6 Biomarker interpretation and translational testing

**FIGURE 2:**
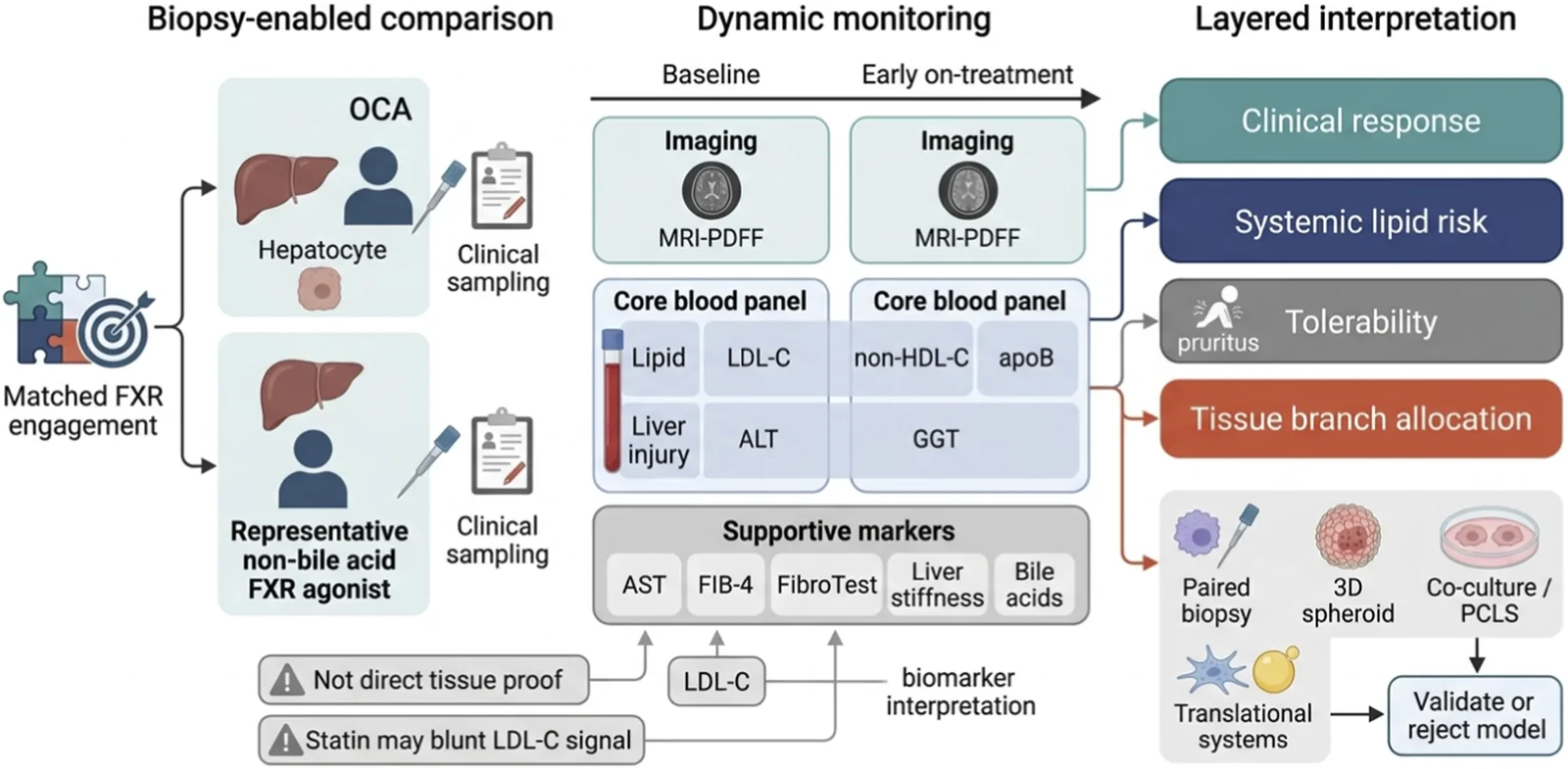
Translational testing workflow for the OCA-centered model. Early-phase comparison should match FXR target engagement and combine imaging, lipid-risk markers, aminotransferases, non-invasive tests, bile-acid pharmacodynamics and, where feasible, tissue branch-allocation readouts. Human multicellular systems should be used to test whether benefit can occur without concordant sterol-stress and inflammatory-fibrogenic signatures.

Biomarkers are most useful here when assigned to layers. The clinical response layer includes histology, MRI-PDFF, ALT, AST, GGT, liver stiffness, FIB-4 and FibroTest. These readouts can track response or risk; in REGENERATE, several non-invasive tests changed in association with OCA response (Rinella et al., 2022). The systemic lipid-risk layer includes LDL-C, non-HDL-C, apoB and LDL particle concentration. These variables matter clinically, but they do not measure intrahepatic free cholesterol. Pruritus and bile-acid or sensory-neuronal symptoms belong to the tolerability layer. Tissue branch allocation requires different readouts: free-cholesterol burden, cholesterol crystals, crown-like structures, macrophage activation, IL-1beta, ACTA2, COL1A1, SLC27A5/FATP5 and ACOX1. A proposed translational testing workflow is shown in Figure 2.

This layering avoids two common mistakes. One is false reassurance: a fall in MRI-PDFF or ALT does not prove that an injurious tissue program is absent. The other is false toxicity assignment: an LDL-C increase or pruritus episode should not automatically be called liver-intrinsic injury. Table 2 grades the confidence of each model node, and Table 3 summarizes biomarker interpretation by layer.

**TABLE 2:** Evidence confidence grading for the OCA-centered model.

Model node	Current evidence base	Evidence level	Human validation status	Main uncertainty/needed validation
OCA histological activity	FLINT and REGENERATE biopsy-based trials	Tier 1: human intervention	Supported	Magnitude and clinical benefit-risk balance remain context dependent
LDL-C/apoB signal	Healthy volunteer, NASH lipoprotein analyses and CONTROL	Tier 1: human intervention	Supported as systemic signal	Does not prove intrahepatic cholesterol retention or crystal formation
Pruritus	OCA trial safety data plus itch receptor biology	Tier 1 for clinical phenotype; mechanistic support from preclinical/human itch studies	Supported as tolerability phenotype	Specific causal pathway for OCA-induced pruritus in MASH remains incompletely assigned
FATP5/SLC27A5 benefit pathway	Fatp5 knockout, bile-acid fatty acid uptake work and OCA humanized-transporter models	Tier 4: preclinical/humanized model	Not validated in treated MASH patients	Requires human target-engagement assay and responder correlation
Cholesterol crystals/crown-like structures	Human MASH pathology and murine resolution studies	Tier 3: human pathology plus preclinical intervention	Supported as MASH pathology, not as OCA-induced human lesion	Requires paired OCA-treated human tissue
OCA sterol-stress liability branch	FXR-dependent hepatotoxicity in fatty liver mice	Tier 4: preclinical	Not directly validated in human liver	Dose, species and exposure translation unclear
ACOX1 modifier	Peroxisomal beta-oxidation biology and OCA-ACOX1 inhibition models	Tier 4: preclinical	Not clinically validated	Driver vs. overload marker remains unresolved
Intestinal FXR/FGF15-19 competing model	Organ-specific FXR, gut-liver bile-acid literature, and scaffold-specific PK/PD studies	Tier 2/3/4 mixed	Partially inferential in MASH drug trials	Requires paired hepatic and intestinal pharmacodynamic readouts, Cmax/AUC, hepatic extraction or uptake efficiency, FGF19, bile-acid profile and portal–peripheral exposure assessment
Layered biomarker interpretation	Clinical trials, NIT analyses and pathology logic	Conceptual synthesis	Operationally testable	Requires prospective biomarker-tissue co-localization

**TABLE 3:** Layered biomarker interpretation and failure conditions.

Layer	Representative readouts	What a positive result supports	What it does not prove	Failure condition for the model
Clinical response layer	Histology, MRI-PDFF, ALT, AST, GGT, FIB-4, FibroTest, liver stiffness	Anti-steatotic, biochemical or fibrosis-risk response to a scaffold	FATP5 dependence or absence of tissue liability	Clinical response occurs without any predicted tissue benefit signature
Systemic lipid-risk layer	LDL-C, non-HDL-C, apoB, LDL particle concentration, statin response	Clinically actionable atherogenic lipoprotein risk signal	Hepatic free-cholesterol retention or cholesterol crystal formation	Plasma lipid signal fails to correlate with any tissue sterol-stress readout
Tolerability layer	Pruritus frequency, severity, discontinuation and bile-acid symptom profile	Dose-limiting tolerability and adherence risk	Hepatotoxicity or tissue branch allocation	Pruritus separates from all liver injury and tissue-liability readouts
Putative benefit tissue layer	SLC27A5/FATP5 abundance, fatty acid uptake, hepatocyte triglyceride load	A scaffold-linked substrate-entry component at clinically relevant exposure	FXR class efficacy or clinical responder selection	FATP5 dependence is absent in human tissue or humanized systems
Candidate sterol-stress branch	Free cholesterol, cholesterol crystals, crown-like structures, macrophage activation, IL1B, ACTA2, COL1A1	Co-localized sterol stress and inflammatory-fibrogenic activation	That LDL-C alone is a sufficient surrogate	Matched hepatic FXR engagement with a non-bile acid agonist produces comparable histological or anti-steatotic benefit without increased intrahepatic free cholesterol, cholesterol crystals, IL1B or ACTA2 in paired tissue
ACOX1 modifier layer	ACOX1 expression/activity, peroxisomal beta-oxidation, redox stress and perturbation response	A context-dependent modifier of the benefit-liability profile	Clinical value of ACOX1 inhibition	ACOX1 behaves only as a passive overload marker without causal effect

The most informative clinical experiment would be narrow and early. It would compare OCA with at least one representative non-bile acid FXR agonist under matched target engagement and with biopsy-enabled sampling where ethically feasible. MET409 is an instructive example because it showed liver-fat reduction with a differentiated short-term profile, but the design principle is not limited to that scaffold (Harrison et al., 2021). Baseline and early on-treatment assessment should pair MRI-PDFF with LDL-C, non-HDL-C, apoB, ALT, GGT and bile-acid composition. Tissue sampling, if justified, should measure free-cholesterol burden, cholesterol crystals or crown-like structures, SLC27A5/FATP5 abundance, ACOX1, IL1B, ACTA2 and COL1A1, ideally with spatial co-localization.

Human multicellular systems would provide a second test. Primary human hepatocytes, hepatocyte-rich spheroids, precision-cut liver slices, liver-on-chip systems and hepatocyte-macrophage-stellate-cell co-cultures can compare OCA with non-bile acid agonists at exposures calibrated to clinically relevant target engagement (Khetani and Bhatia, 2008; Godoy et al., 2013; Kostrzewski et al., 2017; Kozyra et al., 2018; Govaere et al., 2020). Hepatocyte monoculture is not enough for this question. The candidate liability branch depends on macrophage and stellate-cell participation, and reduced hepatocyte triglyceride does not adjudicate the inflammatory-fibrogenic branch.

A minimal experimental package would include a steatotic human hepatocyte-rich system for lipid loading and FATP5-linked fatty acid entry, a multicellular inflammatory-stromal system for macrophage and stellate-cell activation, and a tissue-preserving platform such as precision-cut liver slices when available. Cell-type-specific and spatial readouts would be preferable to bulk expression alone. IL1B or ACTA2 expression is informative, but co-localized free cholesterol, macrophage activation and fibrogenic markers would be more persuasive.

The failure condition should be stated before the experiment begins. If human tissue or humanized systems show comparable anti-steatotic benefit without concordant sterol-stress and inflammatory-fibrogenic signatures, especially with non-bile acid FXR agonists, the OCA-centered model should not be generalized. A concrete rejection criterion would be more stringent: if a non-bile acid FXR agonist with matched hepatic FXR target engagement demonstrates equivalent histological or anti-steatotic benefit but shows no increase in intrahepatic free cholesterol and no upregulation of IL1B or ACTA2 in paired liver biopsies, the OCA-centered sterol-stress branch should be rejected as a generalizable FXR model. It would remain useful as a scaffold-specific lesson, but not as a drug-development algorithm. Table 4 summarizes the practical implications for clinicians, trialists and drug developers.

**TABLE 4:** Practical implications for clinicians, trialists, drug developers and translational researchers.

Audience	What this model helps interpret	What it does not justify	Practical implication
Clinicians	Why LDL-C, pruritus and liver response should be interpreted as different layers	Use of OCA for MASH or inference of hepatic crystals from LDL-C alone	Manage cardiometabolic risk; use NITs for risk stratification; avoid tissue-mechanism claims from blood lipids alone
Clinical trialists	How to design biomarker panels that separate response, lipid risk, tolerability and tissue branch allocation	MRI-PDFF or ALT alone as proof of safety	Prespecify apoB/non-HDL-C, pruritus, bile-acid pharmacodynamics, NITs and tissue readouts when feasible
Drug developers	Why scaffold chemistry, tissue exposure and target engagement matter beyond the target name	FXR class-wide inference from OCA alone	Compare hepatic and intestinal target engagement, bile-acid composition and human multicellular responses across scaffolds
Basic/translational researchers	Which model nodes require direct testing	Generalization of FATP5 or ACOX1 as validated targets	Use humanized transporter systems, precision-cut liver slices and multicellular co-cultures with explicit failure conditions

## 7 Discussion: implications and boundaries

The main implication is developmental rather than therapeutic. OCA should not be used for MASH outside regulatory and guideline-supported indications, and the present evidence does not justify clinical ACOX1 inhibition. The more useful lesson is interpretive. A compound that lowers liver fat is not automatically safe; a compound that raises LDL-C is not automatically producing hepatic cholesterol crystals; and a compound that causes pruritus is not automatically hepatotoxic. These are different biological layers.

The same framing also avoids over-rejecting FXR biology. The OCA program shows that the target is biologically active. It also shows that scaffold, exposure, lipoprotein effects and tolerability can defeat an otherwise plausible benefit-risk argument. A more disciplined conclusion is that FXR remains a pharmacological axis requiring narrower scaffold selection, better biomarker layering and stricter tissue validation.

For clinicians, the model mainly argues against overinterpreting accessible markers. LDL-C, non-HDL-C and apoB should be managed as cardiovascular-risk variables, not as direct tissue-toxicity markers. Pruritus should be handled as tolerability and adherence biology. Non-invasive tests should be used for risk stratification and response monitoring according to MASLD/MASH pathways and guidance documents, not as proof of tissue branch allocation (Kanwal et al., 2021; Rinella et al., 2023b; European Association for the Study of the LiverEuropean Association for the Study of Diabetesand European Association for the Study of Obesity, 2024).

For trialists, the endpoint package needs to be specified before the signal appears. Early-phase FXR studies should not rely on isolated MRI-PDFF or isolated LDL-C. A credible design should include a liver-fat endpoint, an apoB-containing lipoprotein panel, aminotransferases, GGT, bile-acid pharmacodynamics, pruritus and, where feasible, tissue or human multicellular readouts of cholesterol stress and stromal activation. Statin use should be prespecified or stratified, because it can change the systemic lipid signal without resolving the tissue question.

For drug developers, target name is not enough. Scaffold chemistry, tissue exposure, hepatic versus intestinal pharmacodynamics, bile-acid composition and off-target transporter interactions may determine whether an FXR agonist has a usable clinical profile. Approved non-FXR MASH therapies have also raised the bar. Future FXR programs will need to explain why the target adds value and how systemic lipid, tolerability and tissue-risk signals will be monitored.

The model is useful only if it can fail. It predicts that the most concerning tissue pattern would be co-localized sterol stress, macrophage activation and fibrogenic remodeling during OCA exposure, not LDL-C elevation alone. It also predicts that a non-bile acid scaffold may reproduce some clinical benefit without the same tissue branch if exposure and target engagement differ. If those predictions are not supported, the model should be narrowed or discarded.

## 8 Limitations

This article is a hypothesis-driven narrative synthesis, not a systematic review or meta-analysis. It provides no pooled efficacy or safety estimates. Cross-scaffold comparisons remain vulnerable to trial design, dose, duration, population, endpoint architecture, background therapy and tissue sampling. FATP5 and ACOX1 are still mainly preclinical nodes. The cholesterol-stress branch has support from human pathology but has not been validated directly in OCA-treated human liver. Regulatory sources are used here only for approval and program-status facts, not for mechanistic inference. The transition from NAFLD/NASH to MASLD/MASH terminology adds another layer of interpretation, because pivotal studies were reported under older disease names.

These limitations define the boundary of the model. The article should be read as a framework for experimental adjudication, not as clinical practice guidance and not as a class-wide conclusion for FXR agonists.

## 9 Conclusion

OCA does not show that FXR agonists as a class separate efficacy from liver-intrinsic liability. It does provide an unusually informative human scaffold from which a testable pharmacological model can be built. In that model, anti-steatotic benefit may include a putative FATP5/SLC27A5-linked substrate-entry component, whereas the candidate tissue-liability branch is centered on sterol retention, free-cholesterol stress, cholesterol crystals or crown-like structures, IL-1beta-mediated inflammatory amplification and hepatic stellate-cell activation. ACOX1 remains a context-dependent modifier with unresolved causality.

The next step is direct adjudication, not another expansion of narrative evidence. Paired human tissue and human multicellular systems under matched FXR target engagement should determine whether the proposed branches truly separate. If they do, FXR-directed drug development may retain a selective role in MASH. If they do not, OCA will still be instructive: it will show how target-level rationale can fail when scaffold chemistry, tissue exposure and biomarker interpretation are not kept distinct.

## Data Availability

The original contributions presented in the study are included in the article/supplementary material, further inquiries can be directed to the corresponding author.
